# Art therapy’s engagement of brain networks for enduring recovery from addiction

**DOI:** 10.3389/fpsyt.2024.1458063

**Published:** 2025-01-06

**Authors:** Patricia Quinn

**Affiliations:** School of Fine Arts – Graduate Program in Art Therapy, Maharashtra Institute of Technology, Pune, India

**Keywords:** art therapy, addiction, neuroscience, trauma, LSBNs, TBIs, LDs

## Abstract

The field of addiction in its priority to save lives has emphasized harm reduction and medication therapies that have taken precedence over counseling and psychotherapy. The extensive mental health needs, traumatic histories and cognitive challenges of this population call for more availability of all treatments, but also in-depth treatment for the causes of the addiction. The prevalence of trauma is examined with regard to the challenge it presents in treatment for substance use disorder (SUD), and other comorbidities. Two case examples are offered that exemplify how art therapy expedites key information about underlying trauma. Art therapy is proposed as a treatment approach for SUD for its apparent activation of key neural networks that are also impacted by trauma, and its usefulness in engaging those who have cognitive challenges experientially. Quantitative research is cited that suggests art therapy’s activation of the reward system, which may make art therapy useful in treating the stress and inhibition coefficients of addiction that map to neural networks of addiction. The need for additional empirical research is cited that may improve the efficiency and effectiveness of art therapy and mental health treatment.

## Introduction

Currently about 16.5% of the US population has presented for treatment for substance use disorders (SUDs) but only 6% has received treatment (1). As Mojtabai (2) points out the numbers are likely much higher, because of the nature of addiction, and the limited resources available to treat and track SUDs. The highest mortality rates historically have quadrupled since 2002 with 108, 000 deaths in 2022 (3). While the effect of the COVID-19 pandemic and the influx of the synthetic opioid, fentanyl, have contributed to the spike in mortality, this may also be due to the prevalence of untreated co-morbid mental health needs found in many case histories of addiction clients (4). There has also been a reduced emphasis on abstinence as the harm reduction model takes precedence and Medication Assisted Therapy (MAT) is increasingly given in primary care, unaccompanied by counseling or psychotherapy (5).

Art psychotherapy has been found to augment learning (6–8), insight (9, 10) and motivation (11–13) in treatment in ways that enhance verbal therapy (14–16). Those who have been using substances for years or decades have functioned in altered states of consciousness that impact their sense of reality, time, identity and perception that may also encompass post-traumatic dissociation (17). Creativity engages personal history and anchors the client in the present with the challenge of portraying a feeling or experience in some way with chosen materials. Art therapy with its visual and tactile involvement is useful in offering a pathway to visually encoded traumatic memory (18). Helping the client to process their trauma, which may have been lost to declarative memory or verbally inhibited due to high levels of arousal, may reduce its disruptive influence that can span from childhood well into adulthood (19).

Art therapists, (15, 20–24), and colleagues from other mental health fields (25) have found that incorporating art therapy into the individual or group sessions promotes awareness of trauma and reduces denial. When offered in treatment with trauma survivors art therapy promotes more conscious awareness of negative events and their influence on the individual (26), often resolving long standing symptomatology (16). Because of the saliency of visual recall of traumatic events (27), it is logical to assume that large scale brain networks of the default mode and central executive are working together, and are mediated by the salience network in the art making process. The art making absorbs the person’s attention with external planning and organization while associations to what is developing promotes self-reflection. This would account for the art’s revealing content and accompanying emotional responses at times.

There is overlap with these three large scale brain networks with brain regions that have been mapped for addiction that include areas involved with reward, stress, and inhibition (28–30). Brain imaging conducted during art therapy shows activation of nodes in these networks, and therefore may play a role in reconstituting self-regulation, cognition and memory.

People with post-traumatic stress disorder (PTSD) may rely on substances to manage symptoms like intrusive memories, and panic that undermine a sense of self-control. One byproduct of long-term PTSD/SUD is often a reduced capacity for socialization and goal attainment with negative consequences across the life span (31). Art therapy engages clients experientially, and was found by Kaimal and Ray (32), and Kaimal et al. (33) to produce an increased sense of self-efficacy. Integrating trauma therapy with addiction treatment may be a key to reducing PTSD symptoms and thereby improving retention (31, 34). Art therapy may help this combined approach by mediating traumatic memories facilitated by the alpha state induced by drawing as noted by Belkofer et al. (35). They postulated that this more relaxed state may facilitate recall of implicit memory.

Studying people with PTSD with the aid of PET scans showed that verbal recounting of traumatic memory caused more activity in the right side of the amygdala, frontal, temporal and visual cortices (36, 37). This indicates that art therapy may help recover and express memories that are less accessible to verbal centers on the left side of the brain following emotional trauma (38, 39). Recalling and contextualizing these memories helps to understand one’s experience, and mitigate the potential for re-traumatization (40).

A major hurdle in addiction treatment is reducing the euphoric recall of substances during withdrawal cravings that lead to relapse. Art may play a role in the restoration of the brain’s capacity to anticipate natural rewards. Lusebrink (41) noted that art’s sensorimotor properties may work with the cortical networks to revivify motor memory, “including those sequences of motor actions relegated to the basal ganglia” (p. 129). A part of the ventral striatum within the basal ganglia is believed to play a part in valuing the effect of substances of addiction, and incentivizing their habituation (42). To compete with the power of the drug’s sensory-based compulsion that no longer responds well to cognition (43), art making may aid in releasing neurotransmitters that stimulate the dorsal striatum that responds to novel experiences (44). Art’s novel, sensory experiences likely activate the production of acetylcholine which aids in attention and stimulates dopamine release in the dorsal striatum (44) for more natural or goal-directed rewards that do not involve chemicals. This beneficial stimulus of art, if sustained over time, may contribute to pro-recovery decisions after treatment.

For those with SUDs, cognitive communication changes may be caused by even mild traumatic brain injury (TBI) (45, 46). Art therapy has been found to help those with brain injuries, verbal impairments, and learning disabilities that often accompany SUDs to communicate and overcome interpersonal challenges, including those with comorbid PTSD (47–50). Conveyed through the art’s content, the client’s behavior, and verbalizations during and after art making, insights may be obtained that reduce frustration and improve socialization. The attainment of meaningful insight may stimulate reward for improved emotional regulation and cognitive functioning. The art therapy approach would reduce frustration for better impulse control while the experiential, aesthetic, and sensorimotor aspect of art may alter predictive processing (51). This would foster adaptation and promote future experiences of going within using the arts, versus reaching outside of oneself for a mood-altering substance.

## Substance use disorders and their treatment

A substance use disorder (SUD) is defined by the National Institute of Mental Health as a “treatable mental disorder that affects a person’s brain and behavior, leading to their inability, to control their use of substances. People with a SUD may also have other mental health disorders.” [(4), ‘Substance use and co-occurring mental disorders’, para. 1-2]. The Substance Abuse and Mental Health Services Administration (SAMHSA) (1) defines the disorder contextually as preceded by biological, psychological, family and community risk factors. By their count, only 53% of addiction treatment programs assess and/or treat mental health (1, 52). This suggests that the likelihood of mental health co-occurrence is much higher than the estimated 22.8% of those over 18 years old. Identifying and treating the emotional and cognitive needs accompanying or underlying SUDs comprehensively may engage people in more lasting recovery.

The reason for the widespread unacknowledged mental health needs of those with addiction may be explained in part by the competing economic and political forces that shape public funding and perception. The criminalization of addiction since the 1930s has competed with the treatment of addiction as a bio-psychosocial disorder with bulky spending for law enforcement and mass incarceration (53). The introduction of the Minnesota model in the 1950s offered addiction-specific treatment based on Alcoholics Anonymous (54), consisting primarily of group counseling that became the primary model for addiction treatment. Employing para-professionals, usually people with lived experience, in recovery became a necessity as addiction burgeoned during the Viet Nam War era (54). Despite efforts by some states to improve professionalism in SUD treatment, the practice of relying on unlicensed, non-degreed staff for milieu, group and family therapy, continues and this is unlikely to change due to significant workforce shortages (55). Other disparities in the systems of care in the U.S. include the historic lack of integration of addiction medicine in medical and mental health education (56).

Clients report that their most valued element of the treatment experience is the therapeutic alliance with the doctor, counselor or therapist (57). Because engagement with the therapist and extended time in treatment are protective factors for recovery from addiction (58), individualized treatment is important to help clients achieve long-term recovery, including with opioid treatment (59–61). Additionally, one-to-one therapy is also needed to address trauma, which will be discussed in the next section.

### Before and after the covid pandemic

Before the pandemic shuttered many facilities in 2020, only 51% of dedicated addiction treatment facilities were accredited and only 36% overall offered medication to reduce cravings (1, 52). During the pandemic lack of access to medications and poor internet access for telehealth contributed to the increased use of alcohol and illicit drugs (62, 63). Over two-thirds of the record number 108,000 deaths from substances in 2022 were opioid overdose deaths (64, 65). Yet, deaths from opioids and other substances increased steadily *before* the Covid 19 pandemic (66). This suggests that the 12 step programs, free naloxone sprays, and the evidence-based addiction treatments of offering MATs, Cognitive Behavioral Therapy (CBT) and Motivational Enhancement Therapy (MET) alone were not enough to counteract the ensuing addiction crisis.

### The opiate crisis

Efforts to counteract morbidity from opiate overdose since the pandemic have altered approaches to addiction treatment (67, 68). Lifetime MAT maintenance has become the primary treatment protocol for opioid addiction (69, 70) despite a widening treatment gap for young people with regard to MAT initiation and compliance (71). Although these medications help keep people alive, recipients continue to die at a higher rate than the general population (72), and are at risk for developing cognitive problems (73–75). MAT also does not address long term pain sensitivity, hyperalgesia, which can interrupt recovery (73, 76, 77). This includes emotional pain. Thus Park et al. (78) and Johnson and Faraone (69) advocate for a MAT treatment that includes psychotherapy to treat pain and mental health needs to increase successful treatment outcomes. Lack of skilled treatment, drowsiness, and hyperalgesia may contribute to the illicit use of benzodiazepines and alcohol (79), cannabis (80, 81) and other drugs that can undermine MAT adherence, and recovery in general.

## Neurobiological factors of trauma and addiction

Those with adverse childhood experiences (ACEs) are at highest risk of opioid use disorder (82), and MAT non-compliance (83). Early interpersonal traumatic events, like physical and sexual abuse, are strongly associated with numbing, sedating drugs (84–87), and alcohol (19, 88, 89). The SUDs of people with childhood trauma who develop subsequent mental health disorders may be especially unyielding to treatment as it exists today generally (90).

Exposure to parental addiction and mental health problems are prominent risk factors for the development of addiction as parental addiction may undermine attachment and subsequent capacity for connection (91). These negative foundational experiences may result in substance misuse and other risk-taking behaviors, leading to eclipsed educational/vocational opportunities, and poor physical and mental health. These factors can contribute to youths’ criminal justice involvement (92).

The ACE Study (93) revealed a high correlation between trauma and addiction in young adults. Those with ACE scores of four or more were especially vulnerable (94) due to cognitive and emotional deficits impeding healthy attachment and self-regulation and leading to substance misuse (95, 96). Cozolino (97) observed that repeated traumas through childhood appear to lead to maladaptive avoidance styles including addiction, self-harm and personality disorders. Subsequent research has shown that early adversity impacts the neurochemistry and neuro-regulatory functions of the brains of adults resulting in a wide range of mental health problems including addiction (88).

### The neurological changes imposed by trauma

While many people with trauma do not develop addiction, quantitative studies and brain imaging analyses have indicated the high comorbidity of PTSD and addiction in others. Dore et al.’s (98) random study of 253 inpatients for SUDs found 80% had at least one traumatic event and almost 45% screened for PTSD. Results have shown that the symptoms of traumatic stress of anxiety and hyper-vigilance alter brain function in ways that drugs and alcohol can temporarily ameliorate. Bergen-Cico et al. (99) noted that traumatic experiences can alter the endocannabinoid signaling system in the hypothalamus and amygdala which help regulate neuroendocrine responses to stress. A meta-analysis of imaging studies has reported alterations in the PFC and ventral tegmental areas (VTA) for both disorders (100). In addition, brain imaging studies of adults with ACEs show the stress impact of adversity over time Hosseini-Kamkar et al. (101). The areas that primarily contributed to mental health problems in adulthood were increased amygdala reactivity, and reduced lower prefrontal cortex activation. They saw that those with PTSD had reduced activity in the hippocampus, orbital frontal cortex (OFC) insula and striatum. These changes create vulnerability to the stress response that undermines memory, reward processing, inhibitory control and increases risk of addiction.

### The destabilizing effect of substance disorders on the brain

The binge/withdrawal cycle imbalances subcortical neurotransmitter output as the drug’s induced euphoria quickly fades and gives way to the stress of withdrawal. When the hypothalamic-pituitary-adrenal (HPA) system is repeatedly activated by stressors, a chronic condition develops where the sympathetic-adreno-medullary (SAM) axis increases stress-driven cortisol release, resulting in substance-seeking to eliminate the anxiety of withdrawal (102). Reduced connectivity of neural networks results, which extends into periods of abstinence (103). The brain is primarily altered by substances in the following ways:

1. Increased incentive salience of substance use due to craving, vs. goal directed behavior, affecting mnemonic processes and self-regulation (30, 104).
2. Elevated reward -thresholds with decreased reward -function, accompanied by lower motivation and attention, with increased stress response, impulsivity and negative thinking. Repeatedly satisfying cravings leads to structural changes to the nucleus acumbens (NAc). This increases vulnerability to relapse because the long-term potentiation of drug activity reduces the effects of competing stimuli. (30, 103–105).
3. Compromised executive function occurs as the striatal, limbic, and prefrontal cortex (PFC) areas are altered. Increased dynorphin in the nucleus acumbens (NAc) and amygdala (30) lead to dysphoria and negative thinking (104).

Stubbs et al. (106) described a common brain circuit affected in addiction by comparing a large cohort of 1,000 healthy subjects’ brains mapped with functional magnetic resonance imaging (fMRI) to a meta-analysis of 99 studies of the imaging data from a total of 9,047 people with addiction. Subjects with SUDs, which included those affected by alcohol, cocaine, opioids, nicotine and cannabis, showed common connectivity in regions that included the anterior cingulate, bilateral insulae, dorso-lateral prefrontal cortices, and thalamus, but not the medial prefrontal and occipital cortices.

### Adolescent substance use

The effect of repeated drug-taking on the developing prefrontal cortex (PFC) reduces top-down reasoning, leaving youth more likely to transition to a substance use disorder (107). Substances may also alter the adolescent’s developing HPA system functioning, as early binging impairs self-regulation that may continue in adulthood (94, 101, 108, 109). The effects of the most commonly used substances on adolescent brain development suggest significant alterations of areas needed for cognition, emotion and motor function.Alcohol use was found to alter the cerebellum, hippocampus and PFC, (110) and the left temporal lobe, caudate, thalamus and brain stem of adolescents (111). The effect of cannabis on the developing adolescent brain potentially disrupts the endocannabinoid system (ECS) and its physiological regulation (112). The ECS is fundamental to nervous system development, and the function and synaptic neuroplasticity of cognitive and physiological components (113). Using the stimulant methylphenidate for ADHD in early childhood was found to alter the reward system, and result in later depression though the effect may be transient (114).

## Art therapy

In a National Institute of Alcohol Abuse and Alcoholism-funded study by nursing educators, Aletraris et al. (115) found 38.6% of 307 addiction treatment programs offered art therapy, despite their frequent inability to be reimbursed by Medicaid. It was found to be effectively integrated into Motivational and 12-Step approaches. SAMHSA (116) promoted the incorporation of art therapy into group addiction treatment as it was found to engage clients’ abilities, attention and insightfulness. It has also been found to help to identify and manage difficult feelings (24, 26, 117–125). In addition to group therapy, it may be necessary to offer more skilled individual treatment, in order create a confidential space to disclose traumatic experiences. As noted by Hinz (126) group art therapy treatment is usually the norm in SUD programs. This was also seen in this writer’s 23 years of experience with both in- and out-patient care for SUDs. Individualized treatment is needed for a population with such high levels of trauma. An example follows of the usefulness of a brief encounter from the writer’s experience as evening-shift supervisor in an inpatient facility.

A model client, in late middle-age, was leaving without his belongings after one week of a 21-day inpatient, SUD facility at 9:30 PM. He had just learned that he’d been mandated to a two-year half-way house after this treatment. He was distressed, as he had completed several such treatment programs before, alternating with incarcerations due to breaking curfews that were a condition of his parole. He was leaving our facility at the same time as that curfew. He took my suggestion to use art supplies to ‘release stress’ while we discussed his options. He expressed frustration with legal constraints while rhythmically covering the page with a dark blue oil pastel. When he beheld the drawing he was surprised that he’d depicted “the night-time”, identifying, for the first time to himself, he said, his fear of the dark. He briefly referred to a childhood of foster home placements and juvenile detentions. He explained that anxiety at night, when home under curfew, compelled him to go outside and find marijuana. When we identified that this was possibly a phobia that could now be treated, he accepted a psychiatric referral for help with his nyctophobia, completed treatment, and accepted the referral to long term care.

Treating comorbid psychopathology reduces anxiety, hopelessness, depression, and low self-esteem that correlate to poorer outcomes post-treatment as Urbanoski et al. (127) found. The case can be viewed through the schema of the Expressive Therapies Continuum (ETC) (126, 128–130). This model was created to guide the art therapist’s engagement with brain regions via specific art approaches and materials to assess and address clients’ unique presenting needs. The client’s choice of oil pastels and paper offered a sensorial, rhythmic activity with hand pressure and movement to calm the diencephalon and limbic brain from the bottom up, neuro-sequentially (37, 131). The activity appeared to calm the client’s nervous system, which apparently facilitated the client’s recall of personal history, ushered in by the symbolism of the nighttime. He was then able to cognitively (and compassionately) understand his own reactive anxiety, and re-evaluate his choice to leave. The drawing revealed a symptom that he had just experienced and long employed as an avoidant response to some likely trauma, given his childhood of foster homes and juvenile incarceration. Implicit memory appeared to have been stimulated and given shape by art’s sensory experience and graphic qualities. The drawing externalized the nyctophobia so that it could be understood in that context. Information obtained via art’s experiential, sensorimotor activation, as in this case, may have altered his predictive processing regarding his troublesome fear response to nightfall, and the need for substances, which he had tried in vain to abstain from. He would now have the cognitive recall of his long-standing phobia instead, and perhaps gain the therapeutic tools to overcome it. Cognitive realizations such as this may contribute to neuroplasticity (132, 133) for an expanded sense of self, contributing to a more meaningful future orientation.

The externalization of the trauma and its visual apprehension appeared to engage the frontal cortex for a conscious cognition of what was being avoided by the substance use. A more typical example of how processing trauma shifts awareness, stimulating both emotion and interpersonal relating follows. This client was referred for art therapy because she was not making progress in her second week of treatment:

A young mother of four returned to inpatient treatment for the second time in three months and was facing the loss of her children. She presented both times with anxiety, anhedonia and poly-substance use. She barely spoke, and appeared distant in treatment. She had not responded to various psychiatric medication combinations. Meeting with her for the first time, I explained how art therapy can sometimes help to understand the reasons for the use of substances and I invited her to use the materials that included clay, paint, and drawing media to identify anything like that in her life. She chose soft pastel and drew a regressed image of a little girl with a huge tear, and then tearfully abreacted for several minutes. I suggested that she may have been too young to stop the harm, and she related having been abused from the ages of four to 14. I invited her to depict what she would like to have done to be safe or to retaliate, and she drew the same girl, protected by a thick, black line beneath 4 stick figures in hanging nooses. Her affect improved as she thanked me. The next day she related her trauma history in a women’s group. She then interacted well with peers, and completed treatment successfully.

## Art therapy for large scale brain network activation and integration

### Triple network engagement of the DMN, SN and CEN

The aforementioned clients’ patterns of anxiety and multiple relapses contributed to their avoidance of underlying feelings, likely bringing the often reported sense of failure, shame and hopelessness. The therapeutic use of art in a confidential meeting allowed for significant disclosures. Both had experienced significant childhood adversity, and subsequent substance use that contributed to decades of repression or suppression through adulthood. The art making appeared to promote functional connectivity of large-scale brain networks (LSBNs) that process personal history in light of new information which were likely slowed by prolonged addiction (30, 103). This may explain why neither client had previously focused on their traumatic personal history more objectively. This would have depended on connecting the autobiographical information of the default mode network (DMN) to the information gleaned by the central executive network (CEN). These two large networks are mediated by the salience network (SN), which overlaps with both, and contributes to internal communication and decision making, as proposed by Menon’s (134) ‘triple network model’.

### The expressive therapies continuum

The levels of the ETC model represent the bilateral, developmental stages of mental imagery formation and creativity. They are accessed via different art media variables, for example, looser media lending itself to more emotional expression, versus the restrictive media evoking more cognitive expression (126, 135). In this way the ETC model can be used to assess a client’s primary mode of experiencing, and to prescribe art material selections for, among other goals, expression or containment of emotion. This may help those with SUD to manage feelings, and may be useful for looking back autobiographically in a manageable way.

Considering Menon’s model, Lusebrink and Hinz (135) matched the functions of the Expressive Therapy Continuum’s (ETC) components to the brain regions that correspond to the (CEN), (SN) and (DMN). The CEN corresponds to the lateralized, explicit mental (cognitive), emotional (perceptual) and physiological (kinesthetic) responses to stimuli. The DMN, on the other hand, corresponds to the implicit symbolic, affective and sensory responses, with the SN continually switching between the CEN and DMN. In addition to this lateral flow of information, art making combined with the various effects of art materials on each level, appears to support bottom-up and top-down flow, according to the ETC model.

The ETC suggests how art may facilitate a bottom-up approach to trauma treatment that improves effectiveness (131, 136). This may explain how the sensory motor aspect of art engagement overcame the clients’ cognitive barriers through the apparent haptic stimulation of the thalamus and areas in both hemispheres from the peripheral nervous system to the limbic and cortical levels (41, 137). The prompts, materials, and questions the therapist may offer the client are used to scaffold the client to awareness of cognitive, symbolic, affective and perceptual levels.

Internally-focused sensing, feeling and symbolic thought is the domain of the DMN, and SN, while the explicit handling, perceiving, and cognitive operations involved in executing the art is the domain of the CEN. Thus, the activation of the first clients’ sensorimotor, basal ganglia and multimodal association cortices were stimulated by the rhythmic movements of his hand and perhaps the act of “covering up” with the visual depiction of the nighttime engaged both cortical and subcortical areas (12, 41, 135, 138). The triple network activation in the creation of his drawing elicited his personal history in the interpersonal context of art therapy, resulting in cognition of a recovery obstacle likely born of trauma.

The ETC demonstrates how the art can be used prescriptively and intentionally to work on specific levels. This individualizes the treatment, even in a group setting, as the clients’ responses will reveal their own perceptions and predilection for expression. The art offers an isomorphic, symbolic projection (139) of internalized experience that may generate interoception, potentially altering the client’s relationship to their trauma and/or addiction. The ventro-medial PFC and the posterior cingulate cortex are among the brain areas encompassed by the DMN (134, 135). Both are engaged by implicit memory and help to process emotion that may aid in the safe retrieval of trauma history. The seeming sensorimotor activation of these three LSBNs, and their coordination during art therapy may partially explain how clients may be able to revisit traumatic personal history, but remain in homeostasis as has been observed by Hass-Cohen and Clyde Findlay (12), Gantt & Tinnin (140), and Tripp (15).

Reduced connectivity of these networks has been detected in opiate use (141) and in alcohol use (142). Lusebrink and Hinz (135) note that therapeutic art making for certain pathologies may stimulate the salience network in particular. Menon (134) foundthat the SN, which is needed for cognitive functioning, was weakly mapped in the brains of those with addiction, pain, anxiety, and autism. This weakness may cause difficulties with self-monitoring, attention, decision-making and cognition in general, as fronto-temporal or fronto-parietal systems are thrown off (134, as cited in (135).

Beaty et al. (143) note that the CEN and DMN “which can show an antagonistic relationship, actually cooperate during creative cognition and artistic performance.” (p. 87). (135) propose that this may be largely accomplished in art therapy through the SN activation as it facilitates switching attention between the introspective, internally oriented information of the DMN and the externally oriented CEN which is engaged in making creative decisions. Thus, to engage the CEN and DMN together, therapeutic art making may be directed toward better insight, reality testing, and impulse control with prompts that invite consequential and goal-directed thinking to challenge and motivate the client.

### Art therapy and the reward network

As with other rewarding experiences, all psychoactive substances affect the reward system, and as noted, can dysregulate the HPA and SAM systems when individuals enter a binge/reward cycle because of habitual use. In addiction relatively high levels of dopamine (DA) in the ventral tegmental area (VTA) are projected to subcortical areas including the ventral striatum, and to the higher, analytic cortices of the anterior cingulate cortex (ACC), medial prefrontal cortex (MPFC), and orbital frontal cortex (OFC) (105).

The binge/withdrawal cycle alters the reward system and may dysregulate DA expression over time, leading to cortical imbalances that contribute to emotional fluctuation (144). The alteration of cognition, particularly the inability to delay gratification and discriminate between short-term pleasure and the long-term risk of substance dependency is a contributing factor to becoming and staying addicted (81, 102, 144). In the basal ganglia DA impacts declarative and procedural memory (30, 105) as well as motivation, attention, impulse inhibition, and goal directed behaviors, which are all key to overcoming urges to use.

Current treatment protocols may do little to repair reward system functioning. Nestor et al. (103) looked at the brains of 83 subjects abstinent for an average of approximately eight months with fMRI in response to a contingency management (CM) task. CM is a financial incentive approach that is offered by 66% of opioid treatment facilities (145). Even with the monetary reward, subjects showed reduced reward network response and reduced neural connectivity in the whole brain.

The comorbidity between psychoactive substance use and depression is high. Studies have shown as many as 43% of people develop addiction who report lifetime depressions (146), and 59% have been found to develop depression in first month of abstinence of the substance and possibly beyond (147). In treatment and during the following months of post-acute withdrawal, generalized dysphoria and depression may make it difficult to learn and to have a sense of wellbeing (148), putting the addiction client at risk for relapse. This may be in part because the activities and interests of people with addiction can become constricted over time, leaving people in early recovery in a dysthymic state without a source of pleasure (149).

Nieoullon (150) noted that DA is essential to anticipation, preparation and adaptation of behavior. These are key stages needed to shift from a culture of compulsive risk-taking behaviors, to a recovery lifestyle geared toward reducing stress.

### Empirical research in art therapy related to reward network and perception

Biological research by art therapists indicating potential reward network activation and perception is applicable to evaluating art therapy’s benefit for the SUD population. A pilot study using functional near-infrared spectroscopy (fNIRS) revealed activation of the mPFC which is a dopamine target of the reward network (33). In this study, 26 healthy subjects performed three art tasks for a total of 20 minutes. Reward perception was demonstrated when subjects, who were both familiar and not familiar with art, reported improved self-perception and creative confidence.

Han et al. (151) also employed fNIRS technology to compare the effect of different mediums on the brains of 26 healthy subjects based on the ETC model. They found dorso- and ventro-lateral PFC stimulation; areas that contribute to emotional regulation and response inhibition, regardless of the medium selected. These areas, and the OFC activation they found, are also primary nodes impacted in an addiction network mapped by Tolomeo and Yu (29). Furthermore, they recorded activation of the parietal cortex indicating sensory integration and motor judgment. Han et al. (151) also compared the use of traditional art supplies to digital art media, and found similar results, but with lower neural activation for subjects making digital art on the tablet.

Several studies employed functional magnetic resonance imaging (fMRI) to determine areas affected by art-making and art–viewing. Engagement of the mPFC in art making, and other targets of DA’s stress reducing effects (152) were found by Chamberlain et al. (153). Walker et al. (50) used fMRI with military service members with brain injuries and PTSD, and found improved thalamic functioning and an increased sense of belonging among subjects during and after art prompts that focused on community engagement.

Chatterjee and Vartanian (154) using fMRI noted the likelihood of the default mode and reward network engagements in viewing art, and found mPFC, OFC, nucleus acumbens (NAc) as well as insula activation in viewing aesthetically pleasing faces and architecture. And, Lacey et al. (155), using fMRI sets, saw neural connectivity within the reward circuitry of the mPFC, the amygdala, NAc and ventral striatum among subjects viewing art vs. viewing non-art.

Kaimal et al. (156) obtained saliva samples before and after 45 minutes of art-making that showed reduced cortisol levels among 39 healthy adults. Most found the open-ended art-making experience relaxing, and felt it stimulated new learning about themselves, suggesting reward system involvement and art therapy’s potential role in reducing stress.

Another promising study, previously mentioned, indicating reward system engagement, was the quantitative electroencephalogram (qEEG) research of Belkofer et al. (35) which showed the production of alpha rhythms in the brains of artists and non-artists after just 20 minutes of drawing activity. The alpha state is associated with improved mood, self-regulation and memory processes. This may have implications for recall of suppressed memories during creative activity that may bridge implicit and explicit knowledge. Kruk et al. (157) employed qEEG technology to show increased energy in areas of the brain during art making with both drawing and clay that supported memory and meditative processes. The EEG study of King et al. (158) showed increased power in both hemispheres after art tasks, compared to rote motor tasks.

Restoring the reward system can take months or years, after the disruption of the hypothalamic-pituitary-adrenal axis (HPA) from repeated cycles of euphoria and withdrawal (159). As multiple biological research studies show, regardless of variables of medium and time, art therapy may contribute to the restoration of the reward system which may improve cognition, attention, emotional regulation, motivation and a sense of connection and well-being in recovering people.

## Additional advantages of art therapy’s likely effect on the brain

### Remediation of the neural nodes of the addiction network

The widespread addiction network generally includes the amygdala, hippocampus, thalamus, hypothalamus and HPA system, VTA, dorsal striatum and NAc, mPFC, OFC, and the basal and medial forebrain, (28). Stubbs et al. (106) mapped the brain networks for all psychoactive substances of abuse of using MRI and found overlaps with the DMN, SN, and CEN. They also saw atrophy and loss of neurons and connectivity throughout the network in those with long term SUDs. Art therapy research with brain imaging and biochemistry shows therapeutic art making’s apparent stimulation of many of these areas involved with reward processing, cognition and emotion as noted.

### Integrating visual and verbal memory

Since there is almost no dopamine expression toward the perceptual cortex (105) art therapy’s engagement of the perceptual system via visual and tactile art, may contribute to personal memory when the DMN, SN and CEN are also engaged. Yoshii (160) found the thalamus works closely with the retinotectal pathway during fear inducing trauma, and that this may further explain why art therapy, visual biofeedback and bilateral eye movement processes appear to aid in trauma recovery (136, 161).

Areas of the brain most affected by alcohol misuse include the prefrontal cortex & visual-spatial cortices (162–164). Art therapy’s sensory-motor activation and engagement of the visual cortices may therefore help to restore the connectivity and functioning of these areas.

Excessive use of alcohol, as well as PTSD can undermine the expressive and interpretive verbal processing in Broca’s area in the left frontal gyrus and Wernicke’s area in the left temporal gyrus (39, 97, 136, 165, 166). This and other cortical and subcortical changes, especially after repeated traumas, may make cognitive, verbal-only therapies less effective treatments (167). Even in cases where PTSD did not develop, traumatic material was found to reduce verbal recall of these memories (168).

In art therapy, the charged imagery that is symbolically revealed or concealed allows the client to acknowledge and process personal history when they are ready. The therapist’s attunement to, and facilitation of, access to personal history was noted to be felt on a deep level even among those with reactive attachment disorder from extreme neglect and abuse as noted by Klorer (23). This attunement may serve to engage clients on a deeper level and retain them in treatment. Since traumatic memories are somatically based (169) and primarily encoded in the right hemisphere as sense impressions, including images (37, 136, 170–172) art therapy may facilitate opportunities for clients to recall and communicate them. The sensory aspects of art therapy may expedite cognitive and emotional responses to early ACEs because, as Schore writes,”Art therapy is theoretically centered in the affect-laden properties of the visual image, an important potential access point into the child’s inner world” (in 37, p. xii).

### Trauma and stress

Lusebrink and Hinz (135) proposed teaching clients to utilize art as a safe platform for expressing emotional anguish rather than acting on harmful or antisocial urges. To this use of art King and Kaimal (133) wrote, “It is now common knowledge that central nervous system and neurobiological mechanisms of stress response and memory recall are compromised as a result of traumatic experiences and that the creative processes offer an opportunity to rework and repair fragmented memories within an attuned therapeutic relationship” (p. 3). Hass-Cohen (138) suggests one advantage to art therapy for reducing stress is that the ACC, which is part of the SN, and which is activated by conscious experiencing, plays a role in mediating conflicts, and in bridging to higher thinking. The art materials, engaging and anchoring consciousness materially, may help clients externalize negative memories or conflicts which can then be processed metaphorically.

Art’s experiential aspect may account for several quantitative studies validating art therapy’s effectiveness for the treatment of trauma and stress (14, 157, 173–179).

Projective art therapy assessments and prompts may reveal hidden aspects of the client’s pathology for a timely diagnosis of a comorbid mental health condition (180, 181) to improve clients’ success after treatment. Positive experiences may be recalled or anticipated in art therapy prompts as well. Engaging SUD clients’ creativity is generative because art’s spontaneity is the diametric opposite of the repetitious cycles and rituals of addiction (24). The pairing of novel experiences in art with recollections of traumatic memories may reduce reactivity to them (138). Thus, the haptic nature of these novel experiences combined with faithfulness to abstinence, may prime the brains of those recovering from SUDs to tolerate and process negative thoughts, and unexpected events in sobriety.

### Alexithymia

In addition to learning how to manage overwhelming emotions, clients in SUD treatment frequently present with alexithymia, which can lead to relapse (182) as clients use substances to heighten feelings. In a review of the literature, Morie et al. (183) found that alexithymia in addiction clients is connected to loss and reward processing.

Nan and Ho (184) showed that short term art therapy using clay was more effective in treating adults with depression and alexythymia than a general visual art group. In this way, art therapy may compliment Behavioral Action, a goal and activity-based, third-wave CBT approach to depression, that has been shown to stimulate the reward network and can generate new learning to reduce isolation and dysthymia (185).

Suppression of affect may function to isolate negative feelings from early adversities. The visual and tactile stimulation of art may help evoke and process emotional responses in emotionally constricted clients (41, 119, 186). Eliciting the traumatic source of addiction reduces shame (124) and may identify other potential relapse triggers, helping to build a foundation for communication and connection for long-term recovery (187).

### Cognition

Art therapy appears to help people with SUDs to analyze their experiences (126). Lusebrink and Hinz (135) proposed that sequentially organizing one’s art and choosing materials would likely activate the task-oriented functions of the CEN. The cingulate cortex plays a role in discerning and mediating social, emotional and cognitive situations, and is active in memory and visuospatial processing. It is likely engaged in art making when activated by conscious experiencing, planning and sorting as well as anxiety (44). Since the cingulate cortex mediates between the cortex and limbic system (138) this may support reward-based self-regulation, and thus help clients to resist internal and external relapse cues. Dietrich (188) described how art’s cerebral integration can be generalized to other cognitive operations, such as for balanced thinking and behavior: “To that end, prefrontal circuits are involved in making novelty fully conscious, evaluating its appropriateness, and ultimately implementing its creative expression” (p 1023).

### Thalamus function

The thalamus, as the gatekeeper of sensory activation, acts as a relay station. Its engagement of cortical and subcortical areas contributes to functions ranging from circadian rhythms to motor control and cognition. It is presumed to be active during art therapy via visual imagery and tactile handling of art materials (41, 44). Lusebrink (41) noted that the widespread impact of art therapy begins with the somatosensory information processing from the peripheral nervous system to “a specific nucleus in the thalamus and to the somatosensory cortex…” (p. 127). She describes that from there the information is transmitted to, and integrated by, the multi-association area of the parietal lobes, and visual cortex, thus contributing to widespread connectivity.

Addiction network areas that are impacted negatively by the chronic use of substances like cocaine, alcohol, cannabis and opioids include the bilateral thalamus (103, 106). Early trauma that often precedes SUDs can lead to under-activity in the thalamus (29, 189, 190). Krystal and Neumeister (191) found that high levels of arousal from trauma disrupt transmission of sensory information to the limbic system, cingulate and frontal cortices, leading to increased dissociation. These areas overlap with those identified as part of the addiction network and may be particularly pronounced in cases of childhood abuse and neglect (160, 190). Both papers identified smaller thalamic volume in adult clients with significant childhood trauma, while Uhl et al. (30) and Zhai et al. (192) noted smaller thalamus and lower brain activity during visual working memory tasks in adolescents who drank heavily.

### Co-occurring traumatic brain injuries

In the general SUD population, Cermak et al. (166) found a high correlation of childhood TBIs that lowered verbal literacy and fluency. Due to amnesia, people who enter treatment for addiction may not be aware of having a traumatic brain injury. Also, TBI symptoms may be masked by PTSD, as they share many traits, some of which overlap with addiction as well such as depression, mood destabilization and cognitive issues (193). Up to 80% of people with SUDs have an acquired and/or traumatic brain injury that hampers their utilization of addiction treatment with a destabilizing effect on mental health and recovery (194). Kline (48) found art therapy helped people with TBIs to accept neurocognitive changes, and facilitated communication. The projective nature of art expression (195), and stimulation of visual memory may thus help clients recall forgotten brain injuries, and improve communication for these clients in addiction treatment.

Behavioral indicators during art therapy such as excessive frustration, graphic indicators of neurological dysfunction, or tiring easily may generate a brain injury assessment that would improve outcomes for these clients. Revealing the TBI may help these clients prevent re-injury by motivating them to abstain from substances. Using art therapy with military veterans with traumatic brain injuries and PTSD, Walker et al. (16, 50, 196) found art therapy was effective for both disorders concurrently at Walter Reed Hospital’s National Intrepid Centers of Excellence (NICOE) unit in Bethesda, Maryland. As noted, their research found a correlation between visual themes of social connectedness and highlighted improved thalamic functioning in participants.

### Facilitating learning in group treatment

People with attention deficit/hyperactivity disorder (ADHD) and other learning disabilities (LDs), may struggle to focus in psycho-therapy and -education groups that are primarily verbally oriented (197). Including art therapy prompts in clinically and educationally oriented groups may help to engage clients with LDs as well as TBIs. Comorbid learning disabilities are widely overlooked in SUD treatment settings (198). These may range from developmental and intellectual disabilities to those with visual tracking, memory, attention and comprehension problems. Another overlooked sub-group of SUDs, those with intellectual and developmental disabilities may use drugs or alcohol to fit in to reduce anxiety and/or artificially elevate their self-esteem (199). Integrating art into clinical and didactic groups may facilitate engagement and communication with those who verbally struggle in this cohort.

Learning disabilities, such as ADHD are a common precursor to substance misuse and this may in part be due to disruptions in pathways of reward processing, behavioral control and emotional regulation (200, 201). Almost half of those with (ADHD) have been found to have developed a SUD, which is twice as many found in the general population (198, 202–204). ADHD and SUDs show a bi-directional causality as traumatic experiences are believed to be a contributing factor in some cases of ADHD, and ADHD can lead to low self-esteem and risky externalizing behaviors like substance use in youth and adults (200, 205).

Art therapy researchers in Iran (206), have validated an art therapy assessment of graphic indicators for ADHD that also differentiated the graphic traits of other learning disabilities from those of ADHD. Their use of the Formal Elements of Art Therapy (FEATS) (207, 208) assessment showed a differential use of space, depictions of people, and picture organization to be valid indicators of ADHD across several cultures and languages. If adapted for SUD treatment, this assessment may help to identify ADHD comorbidity sooner, as well as other graphic traits archived in the ongoing FEATS graphic indicator research, in order to increase successful completion for many affected adolescent and adult clients.

## Conclusion

In writing about affective neuroscience Panskepp, et al. (209) noted that the brain’s circuitry for play is the same as that for joy and connection, but that this genetically established system needs to be engaged. Though sometimes frustrating at first, the open-ended experience of one’s own creativity often surprises one with insight, and connects people to each other (Hass-Cohen and Clyde Findlay (12). The insight attained can be rewarding (210) and an occasion for joy.

Art therapy has been shown to complement existing addiction treatment models. Trauma-Focused (TF) CBT combined with expressive therapies was found to increase flexibility and effectiveness of TF-CBT (177, 211–213). Gantt and Tinnin’s visually-based trauma narrative (2007) corresponds to narrative therapy with adults, or adolescents (214). Art therapy has been used to increase understanding of spirituality (215–218), and to integrate with other self-help philosophies (118, 124, 126, 219).

Art therapy may engage circuitry for rewarding emotions and insights through behavioral action that brings mastery and self-awareness (220). Art therapeutically integrated with psycho-education would interject a receptive state of playful openness (24, 120) making knowledge about addiction more accessible, especially for those with verbal challenges. Those abused or neglected as children, may safely awaken memories through their art that may alter the clients’ relationship to their SUD. Organizing the art and dividing time and energy to what their hands are doing may mitigate the flooding of emotion that can occur from such revelations. By stimulating and harmonizing areas of the brain possibly undermined by substances, brain injuries or learning disabilities, art therapy may bestow more receptive and expressive abilities. This is especially true for those with low capacity for trust, the marginalized, and those from disadvantaged backgrounds who are most at risk of death from addiction (221) Oepen and Gruber (222).

The SUD client may require a measure of support and privacy for disclosure where trauma is suspected. Schaverien (223) noted that making art during psychotherapy may reduce the client’s self-consciousness by investing their attention partially into their artwork. This may facilitate a relaxed state of reverie through the default mode network to reconnect with important personal history as it works together with the executive center of the brain. This may promote interoception and expression of important clues to the role that addiction has played, as well as its burden on the client and their loved ones. The potential for stimulation of the reward system, which may be altered by long term addiction with a new sensory activity, also makes art therapy a significant primary treatment approach for SUDs.

Mapping art therapy’s activation of critical brain’s areas, as compared to the addiction network, would further ascertain the effectiveness of art therapy for SUD treatment. There is also need for more research into the effect of art making on the brain areas affected by trauma. Longitudinal research into art therapy’s effect on the reward system, and the mediums and methods that may help restore it would contribute to the utilization of protocols like the ETC for assessing and treating various substance and behavioral addictions. Research into the neural basis for the use of art therapy for LDs and TBIs as well as their respective graphic indicators could help identify and treat these problems early for better utilization of services. This information could be life-saving, especially regarding suicidal and gender-non-conforming clients.

For most people entering treatment for addiction, their experience with art is as remote as their understanding of their own psychology. The capacity for change for people with addiction is often slowed by trauma that undermines learning, perception and emotion. Art therapy’s potential for revealing and treating the wide-ranging comorbidities found in people who develop addictions may help SUD treatment to become more effective and economical. Respectful therapeutic encounters with art materials may help people with SUDs understand themselves in profound ways and to experience what it is like to thrive versus survive in a humanistic way.

## Author’s note

The author has worked for 23 years for non-profit addiction treatment centers in the U.S. and is in currently in private practice in the Hudson Valley, NY. She has authored *Art Therapy in the Treatment of Addiction and Trauma* (2021), and is currently working on *The Neuroscience of Art Therapy for Addiction and Trauma.*

## Data Availability

The original contributions presented in the study are included in the article/supplementary material. Further inquiries can be directed to the corresponding author.
